# Frequency and role of NKp46 and NKG2A in hepatitis B virus infection

**DOI:** 10.1371/journal.pone.0174103

**Published:** 2017-03-22

**Authors:** Teppei Yoshioka, Tomohide Tatsumi, Takuya Miyagi, Kaori Mukai, Kumiko Nishio, Akira Nishio, Yoshinobu Yokoyama, Takahiro Suda, Tadashi Kegasawa, Minoru Shigekawa, Hayato Hikita, Ryotaro Sakamori, Tetsuo Takehara

**Affiliations:** Department of Gastroenterology and Hepatology, Osaka University Graduate School of Medicine, Suita, Osaka, Japan; Academia Sinica, TAIWAN

## Abstract

**Background and Aim:**

Natural Killer (NK) cells are involved in the control of viral infection. However, the role of NK cells in chronic hepatitis B (CHB) remains unclear. This study investigated the frequencies and roles of NK cells in CHB, with a focus on activating receptor NKp46 and inhibitory receptor NKG2A.

**Patients/Method:**

Peripheral blood lymphocytes were obtained from 71 CHB patients and 37 healthy subjects (HS). The expressions of NKp46 and NKG2A were analyzed using flow cytometry. The role of NKp46-ligand was assessed using an in vitro co-culture system. Cytotoxicity and IFN-γ production in NK cells were evaluated using RT-PCR and flow cytometry.

**Results:**

CHB patients were classified into treatment-naïve patients with low HBV DNA titer (CHB-L; n = 28), high HBV DNA titer (CHB-H; n = 24) by the cut-off level of serum HBV DNA 4 log copies/ml, and patients receiving nucleos(t)ide analogue (CHB-NA; n = 19). The expressions of NKp46 and NKG2A were higher in CHB-H than in HS/CHB-L/CHB-NA. HepG2.2.15 had higher NKp46-ligand expression than HepG2. When NK cells from HS were co-cultured with HepG2.2.15, inhibition of the NKp46 and NKp46-ligand interaction by anti-NKp46 antibody significantly reduced cytolysis of HepG2.2.15 and IFN-γ production. However, those reductions were not observed in co-culture with HepG2. Additionally, NK cells that highly expressed NKp46 also highly expressed NKG2A (NKp46^high^NKG2A^high^ subset). The frequencies of NKp46^high^NKG2A^high^ subset in CHB-H were higher than those in HS/CHB-L/CHB-NA. Among treatment-naïve CHB patients, the frequencies of NKp46^high^NKG2A^high^ subset were positively correlated with serum ALT (P<0.01, r = 0.45) and HBV DNA (P<0.01, r = 0.59) levels. The expressions of Fas-L, STAT1, TRAIL and CD107a were higher and IFN-γ expression was lower in the NKp46^high^NKG2A^high^ subset than in the other subsets.

**Conclusion:**

The NKp46 and NKp46-ligand interaction contributes to NK cell activation. A novel NK cell subset, the NKp46^high^NKG2A^high^ subset, may be associated with liver injury and HBV replication.

## Introduction

Hepatitis B virus (HBV) infection is a critical cause of liver cirrhosis and hepatocellular carcinoma. HBV has spread worldwide and is a global health problem. The population of patients with HBV infection is estimated at over 300 million [1, 2]. Innate immunity, including natural killer (NK) cells, plays an important role in the control of viral infection [3, 4]. NK cells attack and eradicate infected cells directly in a major histocompatibility complex (MHC)-independent manner [5]. NK cells also play a role in bridging adaptive immunity by producing IFN-γ [6]. In contrast, a previous study demonstrated that activated NK cells suppress HBV-specific CD8^+^ T cells in the human liver, which led to persistent HBV infection by negatively regulating host immunity [7]. Thus, the role of NK cells in HBV infection remains controversial.

The activation of NK cells is controlled by NK cell receptors. Recently, various NK cell receptors were identified and classified into activating and inhibitory receptors [3, 8]. The expressions of NK cell receptors in patients with HBV infection were comprehensively analyzed in several previous reports [9–14]. In regards to activating NK cell receptors, the expression of NKp46 was higher in patients with HBV infection than in healthy control, while the expression of other activating receptors, such as NKp30, NKp44, NKG2C and NKG2D, were not different [12]. NKp46 is a transmembrane glycoprotein [15] and specific NK cell receptor that raises MHC non-restrictive cytotoxicity [16]. The expression of NKp46 is positively correlated with cytotoxicity [17, 18] and plays an important role in eliminating viral infected cells by recognizing viral proteins [19, 20].

In regards to inhibitory NK cell receptors, the expression of NKG2A was lower in patients with HBV infection than in those without HBV infection, while the expression of other inhibitory receptors, such as KIR2DL1/DS1(CD158a/h) and KIR2DL2/DL3 (CD158b), were not different [13]. NKG2A is a major and prominent inhibitory NK cell receptor and is known as lectin superfamily group A [21]. We previously reported that inhibition of NKG2A restored the activity of NK cells from patients with hepatitis C virus (HCV) infection [22]. The expression of NKG2A was associated with clearance of HCV and HBV [23, 24]. These suggest that both NKp46 and NKG2A play important roles in the pathogenesis of viral hepatitis. However, the significance of the expression of NKp46 and NKG2A in HBV patients still remains controversial [9–14]. The roles of NKp46 and NKG2A have not been elucidated in chronic hepatitis B (CHB) and the significance of NK cells co-expression of NKp46 and NKG2A remains unknown.

In this study, we investigated the involvement of NK cell receptors, focusing on NKp46 and NKG2A in the pathogenesis of HBV infection. We evaluated the expression of these receptors and their ligands in CHB patients. Additionally, NK cells with high NKp46 expression also highly expressed NKG2A (NKp46^high^NKG2A^high^ subset) and exhibited higher cytotoxicity and lower IFN-γ production than the other NK cell subset. The frequency of this novel subset was positively correlated with serum ALT and HBV DNA levels. The results of this study improve our understanding of the mechanisms of liver injury and HBV replication.

## Patients and methods

### Study subjects

This study was approved by the Institutional Review Board for Clinical Research at Osaka University Hospital (No. 12238–2) and performed in accordance with the Declaration of Helsinki. Inclusion criteria for this study were age over 20-year-old, absence of known cancer and written informed consent. Seventy-one CHB patients and 37 healthy subjects (HS) were enrolled. The patients with HCV infection were excluded. The CHB patients were divided into 3 groups, treatment-naïve patients with low HBV DNA titer (CHB-L; n = 28), those with high HBV DNA titer (CHB-H; n = 24) and patients receiving nucleos(t)ide analogue (NA) treatment over 24 weeks (CHB-NA: n = 19, 18 with entecavir and 1 with lamivudine/adefovir). The serum HBV DNA levels were assessed by real-time quantitative polymerase chain reaction (TaqMan-PCR) assay. The serum HBV DNA cut-off between CHB-L and CHB-H was 4 log copies/ml. CHB-H were younger and consisted of more HBeAg-positive patients than CHB-L (Table 1). All CHB patients and HS provided written informed consent before enrollment. The clinical data from these participants were given new numbers and anonymized before analysis. All data were provided separately as Supporting Information (S1 Data).

**Table 1:** Background of patients.

	HS	CHB-L	CHB-H	CHB-NA	P-value
Number, n	37	28	24	19	-
Age^†^, year (range)	51.9 ± 15.3 (30–75)	61.1 ± 13.0* (35–78)	43.8 ± 14.3** (25–85)	53.1 ± 12.8 (32–73)	0.0008^#^
Sex, male/female, n	22/15	11/17	12/12	13/6	0.2061^§^
ALT^†^, IU/l	19.1 ± 8.9	22.9 ± 13.4	75.9 ± 107.7***	22.9 ± 15.6	0.0006^#^
HBV DNA^†^, LC/ml	NA	2.7 ± 1.1	6.1 ± 1.8***	1.3 ± 1.2	<0.0001^#^
HBeAg (+/-), n	NA	0/28***	9/15	6/13	0.0003^§^
HBeAb (+/-), n	NA	27/1***	16/8	8/11	<0.0001^§^

HS, healthy subject; CHB-L, treatment-naïve patients with low HBV DNA titer; CHB-H, treatment-naïve patients with high HBV DNA titer; CHB-NA, patients receiving nucleos(t)ide analogue; ALT, alanine aminotransferase; HBV, hepatitis B virus; LC, log copies; NA, not applicable; HBeAg, hepatitis B envelope antigen; HBeAb, hepatitis B virus envelope antibody

^#^; Assessed by Kruskal Wallis test

^§^; Assessed by Fisher’s exact test

^†^; mean±SD

*; P = 0.0118 vs HS

**; P = 0.0125 vs CHB-NA, P = 0.0002 vs CHB-L

***; P < 0.0001 vs the others

### Flow cytometry analysis

Peripheral blood mononuclear cells (PBMC) were isolated using lymphocyte separation solution (Nacalai Tesque, Kyoto, Japan). The PBMC were stained with biotin conjugated anti-human CD56 (B159; BD Bioscience, San Diego, CA), phycoerythrin (PE) anti-human CD3 (UCHT1; BD Bioscience), fluorescein isothiocyanate (FITC) anti-human NKp46 (195314; R&D systems, Minneapolis, MN) and allophycocyanin (APC) anti-human NKG2A (Z199; Beckman Coulter, Fullerton, CA) monoclonal antibodies. The samples were stained with all 4 antibodies simultaneously. Then, the stained PBMC were assessed by flow cytometer using a FACSCanto II (BD Bioscience). Data were analyzed with Flow Jo 7.2.2 software (Tree Star, Ashland, OR).

### Co-culture assay

We used HepG2, a human hepatoblastoma cell line, and HepG2.2.15, which was established from HepG2 by transfecting with a plasmid containing two head-to-tail dimers of the HBV genome [25, 26]. HepG2 was obtained from American Type Culture Collection (Manassas, VA, USA) and HepG2.2.15 was kindly provided by Y. Matsuura (Department of Molecular Virology, Research Institute for Microbial Diseases, Osaka University, Suita, Japan). These cell lines were cultured in a 5% CO_2_ incubator at 37°C. The culture medium was Dulbecco’s modified Eagle’s medium (DMEM; Sigma-Aldrich, MO) supplemented with 10% fetal bovine serum (26140–079; GIBCO, Grand Island, NY).

To examine the cytolysis of NK cells against these target cell lines, a redirected killing assay was performed. NK cells were co-cultured with the target cell lines labeled with carboxyfluorescein succinimidyl ester (CFSE, V12883; Invitrogen, Carlsbad, CA). After incubation for 4 hours, peridinin chlorophyll protein (PerCP) conjugated anti-7-aminoactinomycin D (7-AAD) antibody (420404; BioLegend, San Diego, CA) was added prior to flow cytometry analysis. The rate of specific lysis of target cells was determined as the number of 7AAD^+^CFSE^+^ cells/number of CFSE^+^ cells. The IFN-γ production in the supernatant was evaluated using enzyme-linked immunosorbent assay (ELISA) with a human IFN-γ Quantikine kit (DIF50; R&D systems). Anti-NKp46 neutralizing antibody (5 μl/ml, 9E2; BioLegend) or isotype human IgG1 were added to inhibit NKp46 and NKp46-ligand interaction.

### NKp46-ligand detection in vitro

To detect NKp46-ligand in HepG2 and HepG2.2.15, we used human chimeric antibody, which is fused human NKp46 and Fc region of human IgG1 (1850-NK-025; R&D systems). HepG2 and HepG2.2.15 were incubated with this chimeric antibody for 2 hours. For the secondary antibody, these cells were stained with APC conjugated anti-human IgG1 antibody (97924; R&D systems) for 30 minutes and analyzed by flow cytometry. As negative control, unlabeled human IgG1 (Eureka therapeutics, Emeryville, CA) was used instead of chimeric protein. We also examined the expression of NKp46-ligand in Huh6 and HB611 cell line, kindly provided by K. Ueda (Department of Microbiology, Osaka University Graduate School of Medicine, Suita, Japan). HB611 was established through transfection of HBV genome DNA into the human hepatoma cell line Huh6 and continuously replicates HBV DNA [27].

### Cell sorting and real-time PCR

From freshly isolated PBMC, NK cells were magnetically sorted using a MACS kit (130-092-657, Miltenyi Biotec, Bergisch Gladbach, Germany) according to its instruction. Specific NK cell subsets were electrically sorted using a FACS Aria cell sorter (BD Bioscience). The total RNA was extracted using a RNA micro kit (QIAGEN, Hilden, Germany). From these RNA, complementary DNA were synthesized using reverse transcriptase and ReverTra Ace qPCR Master mix (Toyobo, Osaka, Japan). TaqMan gene expression assays (Applied Biosystems, Foster City, CA) were performed for the following factors: human TRAIL (Assay ID; Hs00366278_m1), Fas ligand (Hs00181225_m1), signal transducer and activator of transcription (STAT)1 (Hs01013996_m1), IFN-γ (Hs00989291_m1) and β-actin (Hs99999903_m1). All of these target gene expression levels were normalized to the human β-actin expression levels.

### Degranulation and intracellular cytokine staining of NK cells

To examine the function of NK cells, isolated PBMC were co-cultured with or without K562, an NK cell-sensitive cell line, at an effector-to-target (E/T) ratio of 1:1. FITC conjugated anti-human CD107a monoclonal antibody (H4A3; BioLegend) was added at the time of co-culture to assess degranulation. After co-culturing for 1 hour, Golgi stop solution (1 μl/ml; BD Bioscience) was added and the cultures were incubated for 4 hours. Then, a fixation/permeabilization solution (554714, 100 μl/sample, BD Bioscience) was added. After fixation, the samples were stained with PE conjugated anti-human IFN-γ monoclonal antibody (25723.11; BD Bioscience), CD56, CD3, NKp46 and NKG2A antibody and analyzed using flow cytometry.

### Statistical analysis

The data are provided as the mean ± SEM or box-and-whisker plots unless otherwise indicated. An unpaired Student’s t-test, the Mann-Whitney U test, a 1-way ANOVA or the Kruskal-Wallis test were applied using JMP software (ver. 10.0.2, SAS Institute Inc., Cary, NC). A two-sided P value <0.05 was considered statistically significant.

## Results

### The frequencies of NK cells and the expressions of NKp46 and NKG2A in HS, CHB-L, CHB-H and CHB-NA

**Fig 1:**
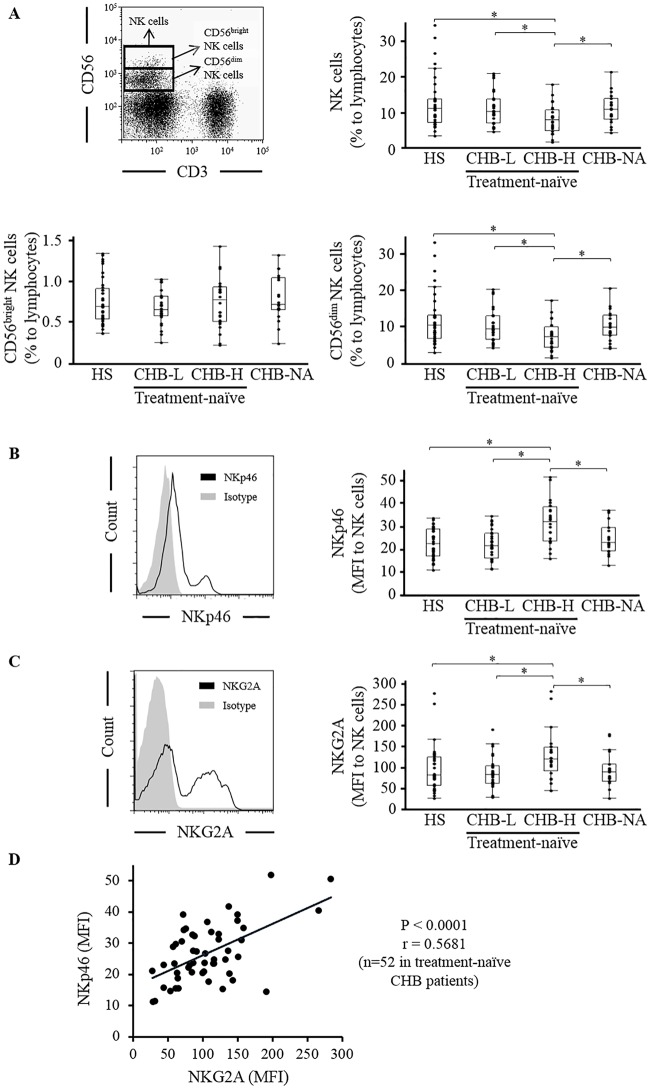
The NK cell frequencies and NKp46 and NKG2A expressions in HS and CHB. The expression of CD3, CD56, NKp46 and NKG2A were analyzed using flow cytometry among 37 HS, 28 CHB-L, 24 CHB-H and 19 CHB-NA. (A) NK cells were defined as CD56^+^CD3^-^ cells on gated lymphocytes and divided into CD56^bright^ and CD56^dim^ NK cells. The frequencies of CD56^+^CD3^-^ NK cells, CD56^bright^ NK cells and CD56^dim^ NK cells were assessed. (B) The expressions of NKp46 and (C) NKG2A on gated NK cells were assessed. (D) Linear regression analysis between the expression of NKp46 and NKG2A was performed in treatment-naïve CHB patients (n = 52). The line represent a regression line. * P <0.05 by Kruskal-Wallis test.

We assessed the proportion of NK cells to lymphocytes in 37 HS, 28 CHB-L, 24 CHB-H and 19 CHB-NA using flow cytometry. CHB-H showed a lower frequency of NK cells than HS, CHB-L and CHB-NA (HS/CHB-L/CHB-H/CHB-NA: 12.7 ± 7.2%/11.1 ± 4.6%/8.2 ± 4.0%/11.1 ±4.3%) (Fig 1A). NK cells were classified into CD56^dim^ and CD56^bright^ subsets according to previous reports [28, 29]. CHB-H showed a lower frequency of CD56^dim^ NK cells than HS, CHB-L and CHB-NA (HS/CHB-L/CHB-H/CHB-NA: 11.9 ± 6.9%/10.4 ± 4.4%/7.4 ± 3.9%) (Fig 1A). There was no significant difference in the frequencies of CD56^bright^ NK cells among HS, CHB-L, CHB-H and CHB-NA (HS/CHB-L/CHB-H/CHB-NA: 0.75 ± 0.28%/0.68 ± 0.21%/0.74 ± 0.30%/0.78 ± 0.28%) (Fig 1A). Next, we analyzed the expression of NKp46 and NKG2A in NK cells. The expression of NKp46 was higher in CHB-H than in HS, CHB-L and CHB-NA (HS/CHB-L/CHB-H/CHB-NA: 22.5 ± 6.5/22.3 ± 6.6/31.7 ± 9.5/24.9 ± 7.1) (Fig 1B). The expression of NKG2A was higher in CHB-H than in HS, CHB-L and CHB-NA (HS/CHB-L/CHB-H/CHB-NA: 96.4 ± 54.3/86.7 ± 38.5/127.2 ± 58.7/93.7 ±40.7) (Fig 1C). Among these 4 groups, CD56^bright^ NK cells displayed higher expression of NKp46 and NKG2A than CD56^dim^ NK cells (S1 Fig). The expression of NKp46 was positively correlated with that of NKG2A, in treatment-naïve CHB patients (n = 52) (Fig 1D). When we divided the expression of NKp46 into NKp46^dim^ and NKp46^bright^ according to a previous report [30], NKp46^bright^ NK cells showed higher expression of NKG2A than NKp46^dim^ NK cells (S2A Fig). The expression of CD107a in NKp46^bright^ NK cells was higher than that in NKp46^dim^ NK cells (S2B Fig). In contrast, the expression of CD107a in NKG2A-positive NK cells was higher than that in NKG2A-negative NK cells (S2C Fig). NKp46 and NKG2A were highly expressed in CHB-H and these receptors might be expressed reciprocally.

### NKp46 and NKp46-ligand interaction activates NK cells

**Fig 2:**
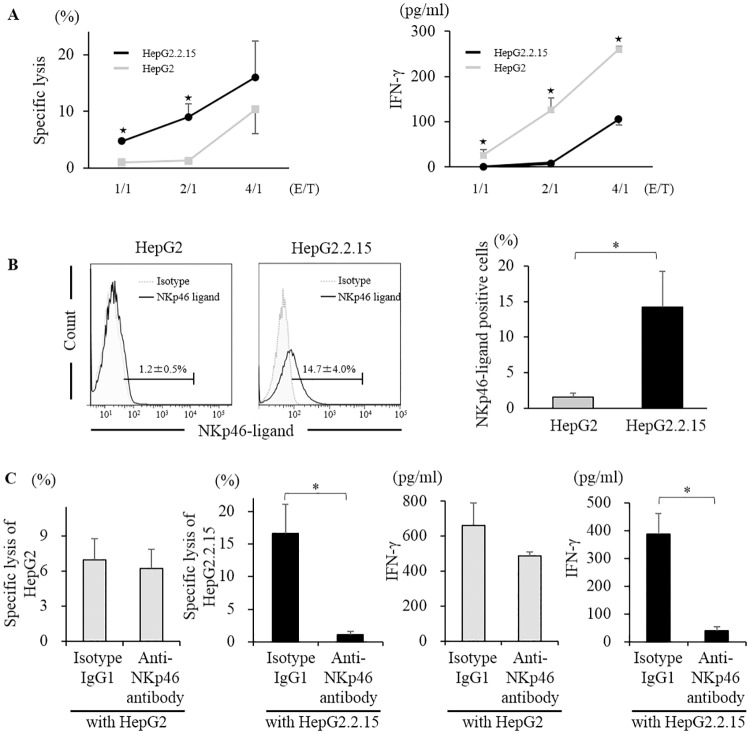
NKp46 and NKp46-ligand were associated with NK cell activation *in vitro*. Purified NK cells from HS were co-cultured with HepG2 (gray line/bar) or HepG2.2.15 (black line/bar). (A) Specific lysis of HepG2 or HepG2.2.15 and IFN-γ in the supernatant were assessed in indicating E/T ratio. ^★^; P<0.05 by unpaired Student’s t-test between HepG2 or HepG2.2.15. (B) NKp46-ligand expression in HepG2 or HepG2.2.15. (C) Specific lysis of HepG2 or HepG2.2.15 and IFN-γ in the supernatant were assessed when neutralizing antibody against NKp46 (Anti-NKp46 antibody) or isotype human IgG1 (Isotype IgG1) were added before co-culturing. *; P<0.05 by Kruskal-Wallis test.

To assess the relationship between NK cells and HBV-infected hepatocytes, we co-cultured NK cells from HS with HepG2.2.15 as a HBV infection model in vitro. HepG2, a parent cell line of HepG2.2.15, was used as a control for target cells. The cytolytic activity of NK cells against HepG2.2.15 was higher than that against HepG2. In contrast, the level of IFN-γ in the supernatant was lower in co-culture with HepG2.2.15 than that with HepG2 (Fig 2A). These results were compatible with functional dichotomy of NK cells, featuring retained or enhanced cytotoxicity and dysfunctional cytokine production by HBV infection [9, 31]. Functional dichotomy of NK cells in viral hepatitis was caused by two different signaling, phosphorylated STAT1 (pSTAT1) related to cytotoxicity and phosphorylated STAT4 (pSTAT4) related to cytokine production. In this co-culture assay, NK cells co-cultured with HepG2.2.15 showed higher expression of pSTAT1 and lower expression of pSTAT4 than those with HepG2 (S3 Fig). Flow cytometric analysis using chimeric antibody fused NKp46 and Fc region of human IgG1, the expression of NKp46-ligand was higher in HepG2.2.15 than in HepG2 (Fig 2B). When we co-cultured NK cells from HS with HepG2.2.15, the addition of neutralizing antibody against NKp46 (Anti-NKp46 antibody) significantly reduced the cytolysis of HepG2.2.15 and IFN-γ production. However, those reductions were not observed in co-culture with HepG2 (Fig 2C). Additionally, we examined the expression of NKp46-ligand in other HBV infection model. The expression of NKp46-ligand was higher in HB611 than in Huh6 (S4 Fig). These data suggest that the difference of NKp46-ligand expression between HepG2 and HepG2.2.15 cell lines affects the function of NK cells.

### Identification of a novel NK cell subset: the NKp46^high^NKG2A^high^ subset

**Fig 3:**
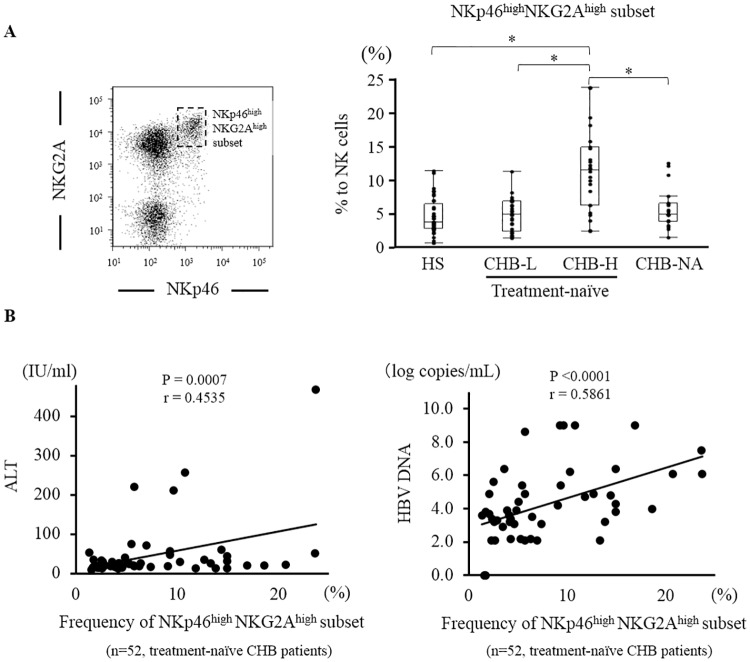
Identification of the NKp46^high^NKG2A^high^ subset and association with clinical data. A unique NK cell subset that is strongly positive for both NKp46 and NKG2A was identified. (A) On gated NK cells, we defined this subset as the NKp46^high^NKG2A^high^ subset (left panel). The frequencies of NKp46^high^NKG2A^high^ subset on gated CD56^+^CD3^-^ NK cells were assessed among 37 HS, 28 CHB-L, 24 CHB-H and 19 CHB-NA (right panel). (B) Linear regression analyses were performed to examine the association between the frequencies of NKp46^high^NKG2A^high^ subset (% to NK cells) and serum ALT or HBV DNA levels in treatment-naïve CHB patients (n = 52). The lines represent regression lines. * P <0.05 by Kruskal-Wallis test.

When we analyzed the expression of NKp46 and NKG2A in NK cells, NK cells that highly expressed NKp46 also highly expressed NKG2A. We identified a unique NK cell subset in which both NKp46 and NKG2A were strongly expressed. We defined this subset as the NKp46^high^NKG2A^high^ subset. The frequency of NKp46^high^NKG2A^high^ subset was significantly higher in CHB-H than in HS, CHB-L and CHB-NA (HS/CHB-L/CHB-H/CHB-NA: 4.8 ± 2.9%/4.9 ± 3.6%/10.3 ± 6.3%/5.8 ± 3.0%) (Fig 3A). We assessed the relationship between the frequency of NKp46^high^NKG2A^high^ subset and clinical data in 52 treatment-naïve CHB patients. The frequencies of NKp46^high^NKG2A^high^ subset were positively correlated with serum ALT (P<0.01, r = 0.45) and HBV DNA levels (P<0.01, r = 0.59) (Fig 3B). Additionally, we divided NK cells other than NKp46^high^NKG2A^high^ subset into NKp46^-^NKG2A^-^, NKp46^-^NKG2A^+^, NKp46^+^NKG2A^-^ and NKp46^+^NKG2A^-^ subset (S5A Fig). There was no statistical difference in the frequencies of these subsets among HS, CHB-L, CHB-H and CHB-NA (S5B Fig). In regards to clinical factors, the frequencies of these NK cell subsets did not show correlation with serum ALT or HBV DNA levels in 52 treatment-naïve CHB patients (S5C Fig).

### Characteristics of the NKp46^high^NKG2A^high^ subset

**Fig 4:**
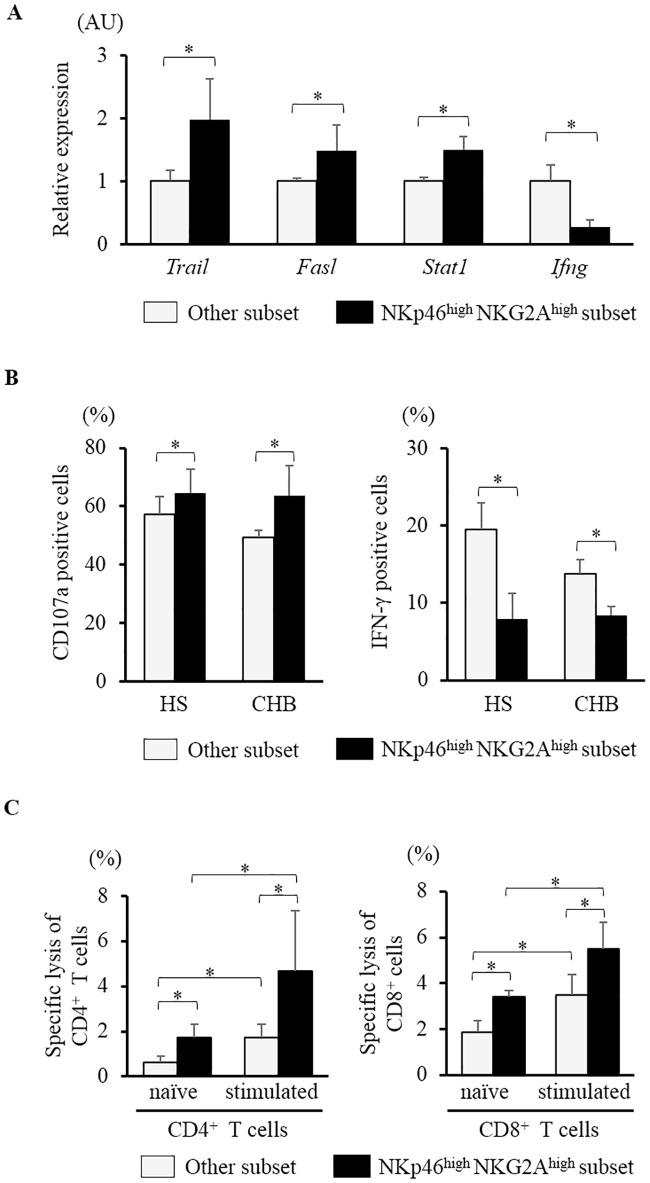
Characteristics of the NKp46^high^NKG2A^high^ subset. NK cells were divided into the NKp46^high^NKG2A^high^ subset and the other subset, which was defined by subtracting the NKp46^high^NKG2A^high^ subset from whole CD56^+^CD3^-^ NK cells. (A) In NK cells from 3 HS, the gene expression levels of the factors related to cytotoxicity and IFN-γ were assessed using RT-PCR. (B) PBMCs from 6 HS and 4 CHB patients were co-cultured with K562. The expressions of CD107a and IFN-γ in the NKp46^high^NKG2A^high^ subset and the other subset were investigated. (C) CD4^+^ or CD8^+^ T cells with or without CD3 and CD28 stimulation were co-cultured with sorted NKp46^high^NKG2A^high^ NK cells or the other NK cells. Specific lysis of CD4^+^ or CD8^+^ T cells were calculated using flow cytometry. * P <0.05 by Kruskal-Wallis test.

To evaluate the role and function of NKp46^high^NKG2A^high^ NK cells, NK cells were sorted into the NKp46^high^NKG2A^high^ subset or the other subset using a FACS Aria cell sorter. The other subset was defined by subtracting the NKp46^high^NKG2A^high^ NK cells from whole CD56^+^CD3^-^ NK cells. The mRNA expression levels of TRAIL, Fas-ligand and STAT1 were higher and that of IFN-γ was lower in the NKp46^high^NKG2A^high^ subset than those in the other subset (Fig 4A). In functional analysis by co-culturing with K562, the expressions of CD107a in the NKp46^high^NKG2A^high^ subset were higher than those in the other subset in both HS and CHB patients. In contrast, the expression of IFN-γ in the NKp46^high^NKG2A^high^ subset was lower than that in the other subset (Fig 4B). The NKp46^high^NKG2A^high^ NK cells demonstrated higher cytotoxic response and lower IFN-γ production than the other NK cells.

Additionally, as NK cells activation was associated with persistent viral infection by the lysis of activated T cells [32, 33], we assessed whether NKp46^high^NKG2A^high^ NK cells could lyse T cells. Human CD4^+^ or CD8^+^ T cells from HS were separated and stimulated with or without anti-CD3 and anti-CD28 antibody for 24 hours. Then, CD4^+^ or CD8^+^ T cells were co-cultured with sorted NKp46^high^NKG2A^high^ NK cells or the other NK cells. The specific lysis of CD4^+^ and CD8^+^ T cells co-cultured with NKp46^high^NKG2A^high^ NK cells were higher than those co-cultured with the other NK cells, with or without CD3 and CD28 stimulation (Fig 4C).

## Discussion

In the present study, we evaluated the frequency and role of NK cells that affect host innate immunity. We assessed the expression of NKp46 and NKp46-ligand in HBV infection and evaluated the role of NK cells, with a focus on a novel NK cell subset, the NKp46^high^NKG2A^high^ subset.

Previous studies have reported the relationship between HBV infection and the expression of various NK cell receptors. Consistent with some studies [10, 12, 13, 24] but in contrast to others [9, 11, 14], we demonstrated that the expression of NKp46 and NKG2A were high in CHB-H. However, few articles have evaluated the balance between activating and inhibitory receptors in NK cells. In this study, multicolor staining on the same sample was performed and, in 52 treatment-naïve CHB patients, the expression of NKp46 was positively correlated with the expression of NKG2A. Our results that the expression of CD107a was higher in NKp46^bright^ NK cells than in NKp46^dim^ NK cells is compatible with the function of NKp46, as an activating receptor. In contrast, the expression of CD107a was higher in NKG2A-positive NK cells than in NKG2A-negative NK cells. These results seem to be incompatible with the function of NKG2A as an inhibitory receptor, however, previous studies reported that NKG2A protects overactivation of NK cells by recognizing human leukocyte antigen E as a ligand [34]. Positive correlation between the expression of NKp46 and NKG2A raise the possibility that the expression of NKG2A negatively regulate NKp46 expressing NK cells with high cytotoxicity.

We showed that NKp46-ligand was expressed in HepG2.2.15 and HB611. The natural ligand of NKp46 is still unknown [19, 20, 35], however, hemagglutinin in the influenza virus was identified as one of the ligands of NKp46 [35]. Therefore, according to previous studies, it is reasonable to assume that one of the mechanisms driving NKp46-ligand is viral infection [16, 36]. Recently, Golden-Mason et al. reported that HCV-infected hepatocytes expressed NKp46-ligand [37]. To the best of our knowledge, the present study is the first report showing NKp46-ligand expression in HBV infection model. Viral infection often leads upregulation of ligands triggering NK cell receptors and these transformation make infected cells highly susceptible to killing by NK cells [38]. We showed that the specific lysis by NK cells in co-culture with HepG2.2.15 was higher than that with HepG2, and inhibition of NKp46 by neutralizing antibody clearly reduced the cytolysis in co-culture with HepG2.2.15 but not in co-culture with HepG2. These results suggest that the interaction of NKp46 and NKp46-ligand was associated with NK cell activity in HBV infection.

Previous reports showed that cytotoxicity was retained or over-activated, whereas the ability of IFN-γ production was hampered in NK cells from patients with viral hepatitis [9, 39–41]. This feature, called functional dichotomy of NK cells in viral hepatitis, related to liver injury and persistent infection. We clearly demonstrated that the NKp46^high^NKG2A^high^ subset has high cytotoxicity and low ability of IFN-γ production. Together with the results that the frequency of NKp46^high^NKG2A^high^ subset was positively correlated with serum ALT and HBV DNA levels, the NKp46^high^NKG2A^high^ subset is associated with liver injury and HBV replication. Tjwa et al. showed anti-viral therapy restored IFN-γ production and that functional changes in NK cells were paralleled by the expression of NKG2A [42]. Our results also showed CHB-NA exhibited lower frequency of NKp46^high^NKG2A^high^ subset than CHB-H. We could not clarify whether the NKp46^high^NKG2A^high^ subset was cause or consequence. However, together with high cytotoxicity and low ability to produce IFN-γ in NKp46^high^NKG2A^high^ NK cells, the alternation of the frequency of NKp46^high^NKG2A^high^ subset is compatible with functional dichotomy of NK cells in HBV infection. Additionally, Peppa et al. showed that activated NK cells deleted HBV-specific CD8^+^ T cells, resulting in persistent HBV infection via TRAIL expression [7]. Our results also showed that the NKp46^high^NKG2A^high^ subset, which possesses high cytotoxicity, injured CD4^+^ and CD8^+^ T cells. Moreover, the cytolysis of CD4^+^ and CD8^+^ T cells with CD3 and CD28 stimulation were higher than those without stimulation. In addition to low IFN-γ production in NKp46^high^NKG2A^high^ NK cells, these results supported that the frequency of NKp46^high^NKG2A^high^ subset is associated with HBV replication.

One limitation in the present study was that we only analyzed PBMC but not liver infiltrating lymphocytes. Kramer et al. demonstrated that the expression of NKp46^high^ NK cells was higher in liver infiltrating lymphocytes than in peripheral blood [30]. This result suggested the expression of NK cell receptors might different between PBMC and liver. Therefore, the expression of NKp46^high^NKG2A^high^ NK cells in liver infiltrating lymphocytes should be assessed in the future to know more detail of this subset.

## Conclusion

In the present study, we showed that NKp46 and NKp46-ligand expression were high on NK cells and hepatocytes in HBV infection, respectively. NKp46 and NKp46-ligand interaction might contribute to the activation of NK cells in HBV infection. A unique NK cell subset, NKp46^high^NKG2A^high^ NK cells, exhibited high cytotoxicity and low IFN-γ production. This subset was associated with liver injury and HBV replication as well as a positive correlation with serum ALT and HBV DNA levels. Future analysis of mechanism regulating the NKp46^high^NKG2A^high^ subset may have potential for new understanding and therapeutic targets of HBV infection.

## Supporting information

S1 Data All data of the manuscript. (XLSX)

S1 Fig The expression of NKp46 and NKG2A in CD56^bright^ and CD56^dim^ NK cells. Among 108 patients consisted of 35 HS, 28 CHB-L, 24 CHB-H and 19 CHB-NA, the expression of NKp46 and NKG2A in each of CD56^bright^ and CD56^dim^ NK cells were analyzed. *; P <0.05.
(TIF)

S2 Fig Association between the expression of NKp46 or NKG2A and cytotoxicity. (A) The expression of NKp46 were classified into NKp46^dim^ and NKp46^bright^ NK cells (left panel). The expression of NKG2A positive NK cells gated on NKp46^dim^ and NKp46^bright^ NK cells were assessed in HS (right panel). (B) The CD107a expression was assessed by co-culture NK cells from HS (n = 6) with K562. The CD107a expression gated on NKp46^dim^ and NKp46^bright^ NK cells or (C) NKG2A negative and positive NK cells were assessed. *; P <0.05.
(TIF)

S3 Fig The expression of pSTAT1 and pSTAT4 in NK cells co-cultured with HepG2 or HepG2.2.15. NK cells were isolated from peripheral blood of healthy subjects using MACS kit (130-092-657, Miltenyi Biotec, Germany). These NK cells were co-cultured with HepG2 or HepG2.2.15 at effector to target ratio of 1;1. After co-culturing for 4 hours, NK cells were stained with CD3 and CD56 monoclonal antibody. After staining, methanol (100μl/well, 15 minutes) and a fixation/permeabilization solution (554714, BD Bioscience, 100μl/well, 15 minutes) were added. After fixation, the samples were stained with anti-human pSTAT1 and pSTAT4 monoclonal antibody and analyzed using flow cytometry. *; P <0.05.
(TIF)

S4 Fig The expression of NKp46-ligand in Huh6 and HB611. The expression of NKp46-ligand in Huh6 and HB611 were analyzed by flow cytometry. The method was mentioned in Patients and method. *; P <0.05.
(TIF)

S5 Fig The association between the frequencies of NK cell subsets and clinical data. (A) CD56^+^CD3^-^ NK cells were classified into NKp46^high^NKG2A^high^, NKp46^-^NKG2A^-^, NKp46^+^NKG2A^-^, NKp46^-^NKG2A^+^ and NKp46^+^NKG2A^+^ subset. The borderline of NKp46 was determined by isotype control (as shown in S2A Fig.). (B) The frequencies of NKp46^-^NKG2A^-^, NKp46^+^NKG2A^-^, NKp46^-^NKG2A^+^ and NKp46^+^NKG2A^+^ subset were assessed among 108 patients consisted of 35 HS, 28 CHB-L, 24 CHB-H, 19 CHB-NA. (C) Linear regression analysis between the frequencies of these NK cell subsets and serum ALT or HBV DNA levels. The lines represent regression lines.
(TIF)

## References

[1] Perz JF, Armstrong GL, Farrington LA, Hutin YJ, Bell BP. The contributions of hepatitis B virus and hepatitis C virus infections to cirrhosis and primary liver cancer worldwide. J Hepatol. 2006;45(4):529–38. 10.1016/j.jhep.2006.05.013 16879891

[2] Organiztion WH.. Department of Communicable Disease Surveillance and Response. Hepatitis B (WHO/CDS/CSR/LYO/2002.2). 2002.

[3] Vivier E, Raulet DH, Moretta A, Caligiuri MA, Zitvogel L, Lanier LL, et al Innate or adaptive immunity? The example of natural killer cells. Science. 2011;331(6013):44–9. 10.1126/science.1198687 21212348 PMC3089969

[4] Nishio A, Tatsumi T, Nawa T, Suda T, Yoshioka T, Onishi Y, et al CD14(+) monocyte-derived Galectin-9 induces NK cell cytotoxicity in chronic hepatitis C. Hepatology. 2016. 10.1002/hep.28847 27640362

[5] Raulet DH. Missing self recognition and self tolerance of natural killer (NK) cells. Semin Immunol. 2006;18(3):145–50. 10.1016/j.smim.2006.03.003 16740393

[6] Lee SH, Miyagi T, Biron CA. Keeping NK cells in highly regulated antiviral warfare. Trends Immunol. 2007;28(6):252–9. 10.1016/j.it.2007.04.001 17466596

[7] Peppa D, Gill US, Reynolds G, Easom NJ, Pallett LJ, Schurich A, et al Up-regulation of a death receptor renders antiviral T cells susceptible to NK cell-mediated deletion. J Exp Med. 2013;210(1):99–114. 10.1084/jem.20121172 23254287 PMC3549717

[8] Tatsumi T, Takehara T. Impact of natural killer cells on chronic hepatitis C and hepatocellular carcinoma. Hepatol Res. 2016;46(5):416–22. 10.1111/hepr.12619 26574168

[9] Oliviero B, Varchetta S, Paudice E, Michelone G, Zaramella M, Mavilio D, et al Natural killer cell functional dichotomy in chronic hepatitis B and chronic hepatitis C virus infections. Gastroenterology. 2009;137(3):1151–60, 60.e1–7. 10.1053/j.gastro.2009.05.047 19470388

[10] Zhao J, Li Y, Jin L, Zhang S, Fan R, Sun Y, et al Natural killer cells are characterized by the concomitantly increased interferon-γ and cytotoxicity in acute resolved hepatitis B patients. PLoS One. 2012;7(11):e49135 10.1371/journal.pone.0049135 23133672 PMC3486810

[11] Micco L, Peppa D, Loggi E, Schurich A, Jefferson L, Cursaro C, et al Differential boosting of innate and adaptive antiviral responses during pegylated-interferon-alpha therapy of chronic hepatitis B. J Hepatol. 2013;58(2):225–33. 10.1016/j.jhep.2012.09.029 23046671

[12] Li W, Jiang Y, Wang X, Jin J, Qi Y, Chi X, et al Natural Killer p46 Controls Hepatitis B Virus Replication and Modulates Liver Inflammation. PLoS One. 2015;10(8):e0135874 10.1371/journal.pone.0135874 26291078 PMC4546267

[13] Bonorino P, Ramzan M, Camous X, Dufeu-Duchesne T, Thélu MA, Sturm N, et al Fine characterization of intrahepatic NK cells expressing natural killer receptors in chronic hepatitis B and C. J Hepatol. 2009;51(3):458–67. 10.1016/j.jhep.2009.05.030 19596474

[14] Li Y, Wang JJ, Gao S, Liu Q, Bai J, Zhao XQ, et al Decreased peripheral natural killer cells activity in the immune activated stage of chronic hepatitis B. PLoS One. 2014;9(2):e86927 10.1371/journal.pone.0086927 24520324 PMC3919705

[15] Pessino A, Sivori S, Bottino C, Malaspina A, Morelli L, Moretta L, et al Molecular cloning of NKp46: a novel member of the immunoglobulin superfamily involved in triggering of natural cytotoxicity. J Exp Med. 1998;188(5):953–60. 9730896 10.1084/jem.188.5.953 PMC3207313

[16] Koch J, Steinle A, Watzl C, Mandelboim O. Activating natural cytotoxicity receptors of natural killer cells in cancer and infection. Trends Immunol. 2013;34(4):182–91. 10.1016/j.it.2013.01.003 23414611

[17] Sivori S, Vitale M, Morelli L, Sanseverino L, Augugliaro R, Bottino C, et al p46, a novel natural killer cell-specific surface molecule that mediates cell activation. J Exp Med. 1997;186(7):1129–36. 9314561 10.1084/jem.186.7.1129 PMC2211712

[18] Moretta A, Bottino C, Vitale M, Pende D, Cantoni C, Mingari MC, et al Activating receptors and coreceptors involved in human natural killer cell-mediated cytolysis. Annu Rev Immunol. 2001;19:197–223. 10.1146/annurev.immunol.19.1.197 11244035

[19] Mandelboim O, Lieberman N, Lev M, Paul L, Arnon TI, Bushkin Y, et al Recognition of haemagglutinins on virus-infected cells by NKp46 activates lysis by human NK cells. Nature. 2001;409(6823):1055–60. 10.1038/35059110 11234016

[20] Arnon TI, Achdout H, Lieberman N, Gazit R, Gonen-Gross T, Katz G, et al The mechanisms controlling the recognition of tumor- and virus-infected cells by NKp46. Blood. 2004;103(2):664–72. 10.1182/blood-2003-05-1716 14504081

[21] Borrego F, Ulbrecht M, Weiss EH, Coligan JE, Brooks AG. Recognition of human histocompatibility leukocyte antigen (HLA)-E complexed with HLA class I signal sequence-derived peptides by CD94/NKG2 confers protection from natural killer cell-mediated lysis. J Exp Med. 1998;187(5):813–8. 9480992 10.1084/jem.187.5.813 PMC2212178

[22] Jinushi M, Takehara T, Tatsumi T, Kanto T, Miyagi T, Suzuki T, et al Negative regulation of NK cell activities by inhibitory receptor CD94/NKG2A leads to altered NK cell-induced modulation of dendritic cell functions in chronic hepatitis C virus infection. J Immunol. 2004;173(10):6072–81. 15528343 10.4049/jimmunol.173.10.6072

[23] Golden-Mason L, Bambha KM, Cheng L, Howell CD, Taylor MW, Clark PJ, et al Natural killer inhibitory receptor expression associated with treatment failure and interleukin-28B genotype in patients with chronic hepatitis C. Hepatology. 2011;54(5):1559–69. 10.1002/hep.24556 21983945 PMC3206734

[24] Li F, Wei H, Gao Y, Xu L, Yin W, Sun R, et al Blocking the natural killer cell inhibitory receptor NKG2A increases activity of human natural killer cells and clears hepatitis B virus infection in mice. Gastroenterology. 2013;144(2):392–401. 10.1053/j.gastro.2012.10.039 23103614

[25] Sells MA, Chen ML, Acs G. Production of hepatitis B virus particles in Hep G2 cells transfected with cloned hepatitis B virus DNA. Proc Natl Acad Sci U S A. 1987;84(4):1005–9. 3029758 10.1073/pnas.84.4.1005 PMC304350

[26] Zhao R, Wang TZ, Kong D, Zhang L, Meng HX, Jiang Y, et al Hepatoma cell line HepG2.2.15 demonstrates distinct biological features compared with parental HepG2. World J Gastroenterol. 2011;17(9):1152–9. 10.3748/wjg.v17.i9.1152 21448419 PMC3063907

[27] Tsurimoto T, Fujiyama A, Matsubara K. Stable expression and replication of hepatitis B virus genome in an integrated state in a human hepatoma cell line transfected with the cloned viral DNA. Proc Natl Acad Sci U S A. 1987;84(2):444–8. 3025872 10.1073/pnas.84.2.444 PMC304224

[28] Cooper MA, Fehniger TA, Caligiuri MA. The biology of human natural killer-cell subsets. Trends Immunol. 2001;22(11):633–40. 11698225 10.1016/s1471-4906(01)02060-9

[29] Caligiuri MA. Human natural killer cells. Blood. 2008;112(3):461–9. 10.1182/blood-2007-09-077438 18650461 PMC2481557

[30] Krämer B, Körner C, Kebschull M, Glässner A, Eisenhardt M, Nischalke HD, et al Natural killer p46High expression defines a natural killer cell subset that is potentially involved in control of hepatitis C virus replication and modulation of liver fibrosis. Hepatology. 2012;56(4):1201–13. 10.1002/hep.25804 22532190

[31] Mondelli MU, Oliviero B, Mele D, Mantovani S, Gazzabin C, Varchetta S. Natural killer cell functional dichotomy: a feature of chronic viral hepatitis? Front Immunol. 2012;3:351 10.3389/fimmu.2012.00351 23420385 PMC3572686

[32] Waggoner SN, Cornberg M, Selin LK, Welsh RM. Natural killer cells act as rheostats modulating antiviral T cells. Nature. 2012;481(7381):394–8. 10.1038/nature10624 PMC3539796 22101430

[33] Lang PA, Lang KS, Xu HC, Grusdat M, Parish IA, Recher M, et al Natural killer cell activation enhances immune pathology and promotes chronic infection by limiting CD8+ T-cell immunity. Proc Natl Acad Sci U S A. 2012;109(4):1210–5. 10.1073/pnas.1118834109 22167808 PMC3268324

[34] Lee N, Llano M, Carretero M, Ishitani A, Navarro F, López-Botet M, et al HLA-E is a major ligand for the natural killer inhibitory receptor CD94/NKG2A. Proc Natl Acad Sci U S A. 1998;95(9):5199–204. 9560253 10.1073/pnas.95.9.5199 PMC20238

[35] Achdout H, Meningher T, Hirsh S, Glasner A, Bar-On Y, Gur C, et al Killing of avian and Swine influenza virus by natural killer cells. J Virol. 2010;84(8):3993–4001. 10.1128/JVI.02289-09 20130050 PMC2849486

[36] Kruse PH, Matta J, Ugolini S, Vivier E. Natural cytotoxicity receptors and their ligands. Immunol Cell Biol. 2014;92(3):221–9. 10.1038/icb.2013.98 24366519

[37] Golden-Mason L, Stone AE, Bambha KM, Cheng L, Rosen HR. Race- and gender-related variation in natural killer p46 expression associated with differential anti-hepatitis C virus immunity. Hepatology. 2012;56(4):1214–22. 10.1002/hep.25771 22505144 PMC3458134

[38] Bottino C, Castriconi R, Moretta L, Moretta A. Cellular ligands of activating NK receptors. Trends Immunol. 2005;26(4):221–6. 10.1016/j.it.2005.02.007 15797513

[39] Ahlenstiel G, Titerence RH, Koh C, Edlich B, Feld JJ, Rotman Y, et al Natural killer cells are polarized toward cytotoxicity in chronic hepatitis C in an interferon-alfa-dependent manner. Gastroenterology. 2010;138(1):325–35.e1–2. 10.1053/j.gastro.2009.08.066 19747917 PMC2862622

[40] Miyagi T, Takehara T, Nishio K, Shimizu S, Kohga K, Li W, et al Altered interferon-alpha-signaling in natural killer cells from patients with chronic hepatitis C virus infection. J Hepatol. 2010;53(3):424–30. 10.1016/j.jhep.2010.03.018 20554341

[41] Zhang Z, Zhang S, Zou Z, Shi J, Zhao J, Fan R, et al Hypercytolytic activity of hepatic natural killer cells correlates with liver injury in chronic hepatitis B patients. Hepatology. 2011;53(1):73–85. 10.1002/hep.23977 21254163 PMC3767982

[42] Tjwa ET, van Oord GW, Hegmans JP, Janssen HL, Woltman AM. Viral load reduction improves activation and function of natural killer cells in patients with chronic hepatitis B. J Hepatol. 2011;54(2):209–18. 10.1016/j.jhep.2010.07.009 21095036

