# Supplementary figures and images for: Frequency and role of NKp46 and NKG2A in hepatitis B virus infection
Source: PLoS One. 2017 Mar 22;12(3):e0174103. doi: 10.1371/journal.pone.0174103 (PMC5362099; doi:10.1371/journal.pone.0174103)

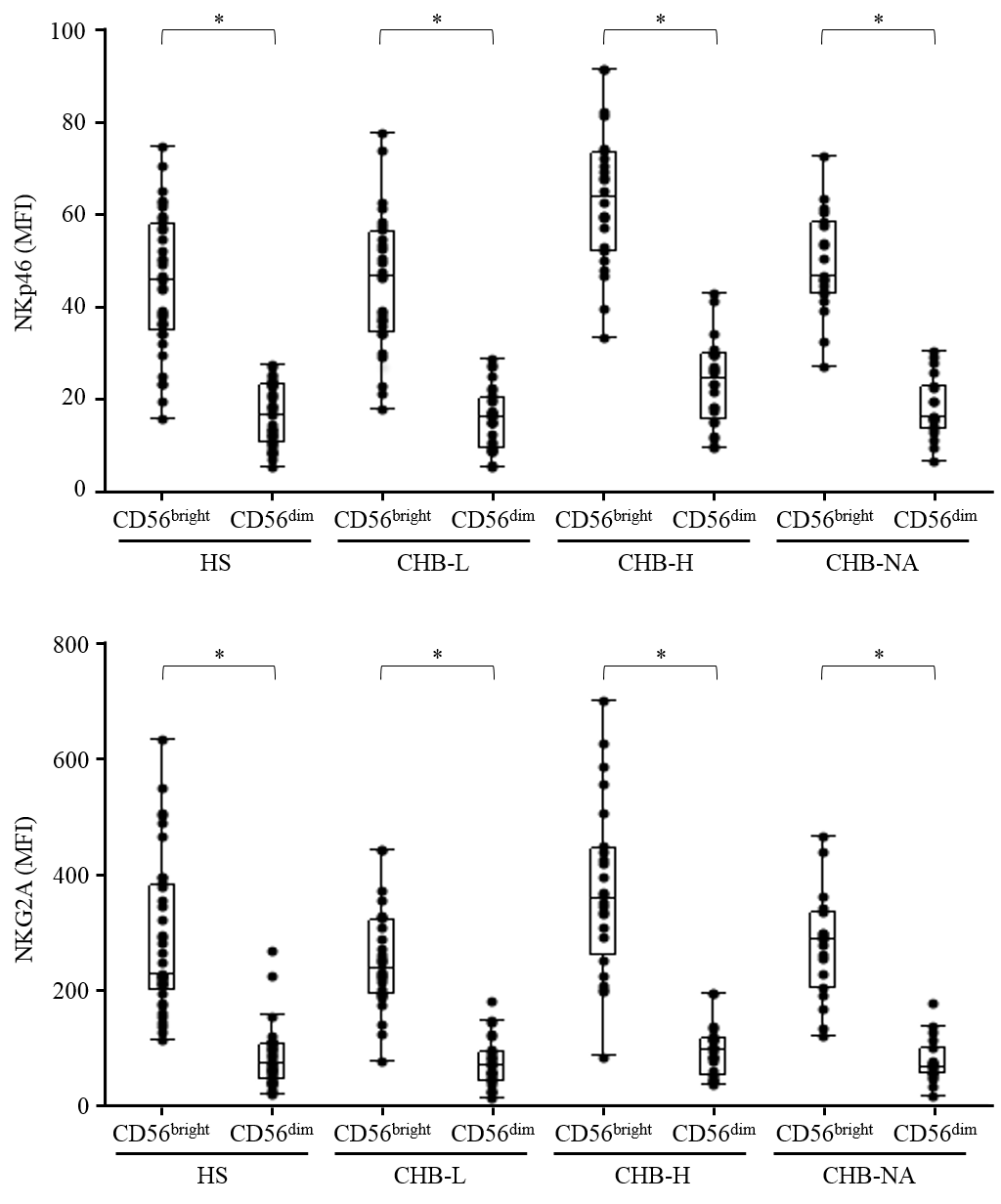

Supplement: S1 Fig — Among 108 patients consisted of 35 HS, 28 CHB-L, 24 CHB-H and 19 CHB-NA, the expression of NKp46 and NKG2A in each of CD56bright and CD56dim NK cells were analyzed. *; P <0.05. (TIF) [file pone.0174103.s002.tif]

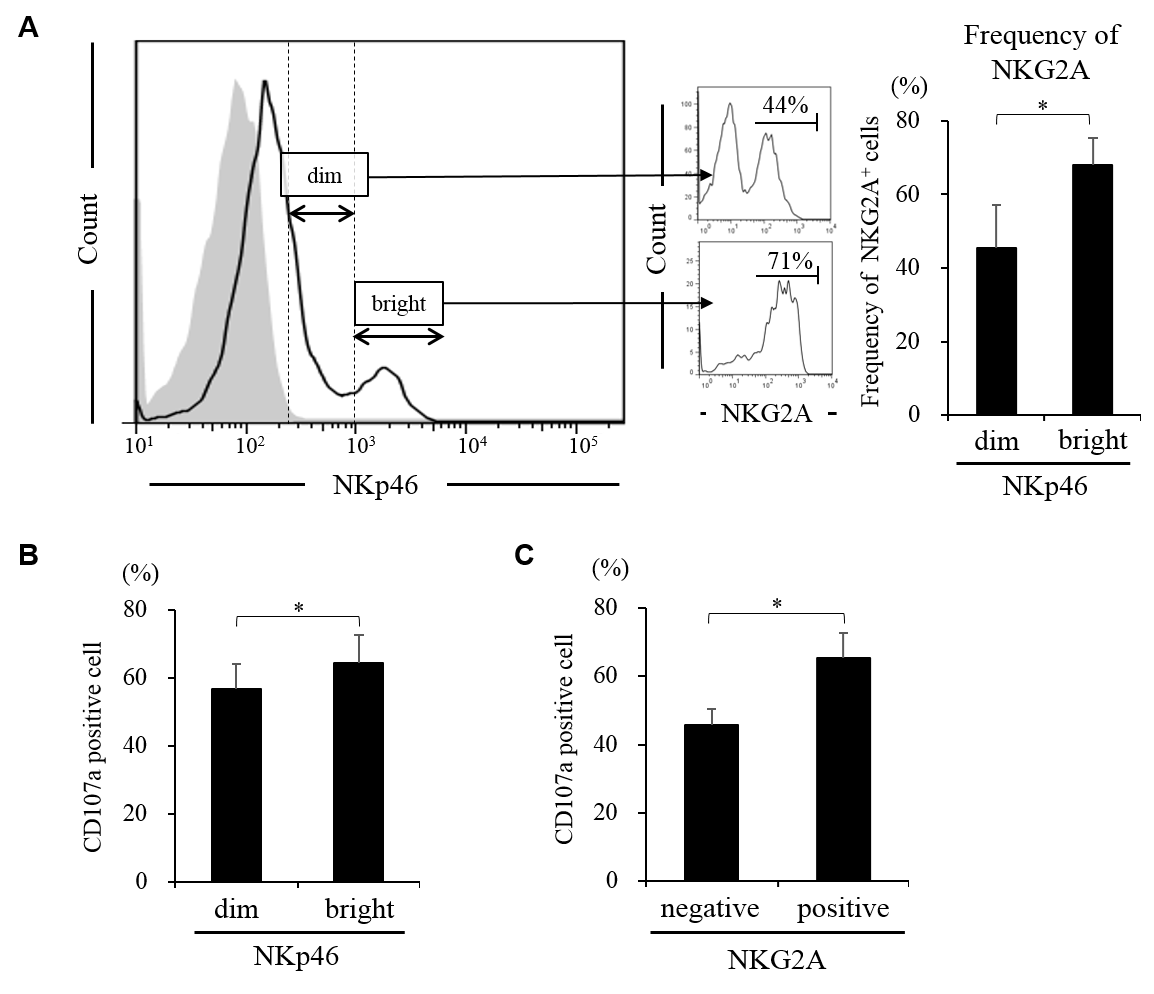

Supplement: S2 Fig — (A) The expression of NKp46 were classified into NKp46dim and NKp46bright NK cells (left panel). The expression of NKG2A positive NK cells gated on NKp46dim and NKp46bright NK cells were assessed in HS (right panel). (B) The CD107a expression was assessed by co-culture NK cells from HS (n = 6) with K562. The CD107a expression gated on NKp46dim and NKp46bright NK cells or (C) NKG2A negative and positive NK cells were assessed. *; P <0.05. (TIF) [file pone.0174103.s003.tif]

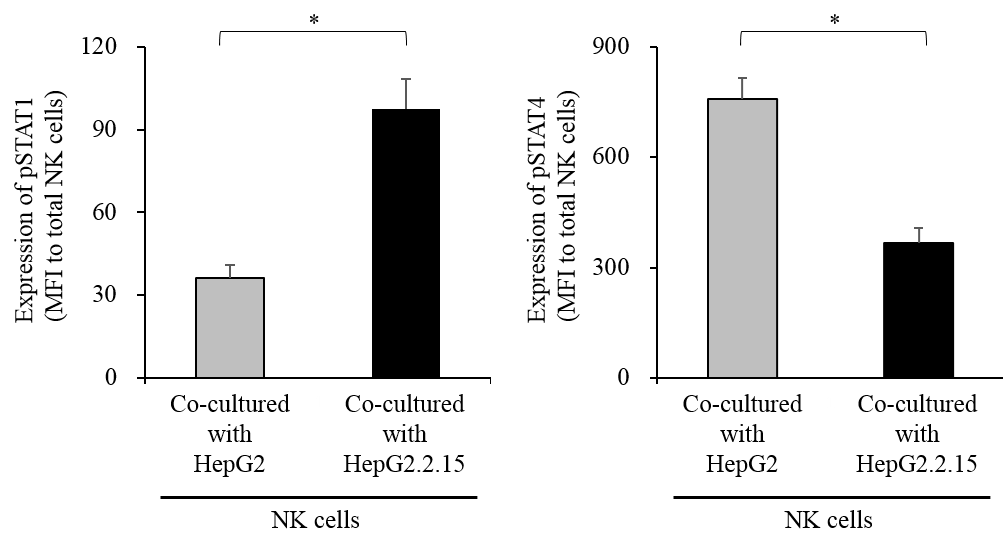

Supplement: S3 Fig — NK cells were isolated from peripheral blood of healthy subjects using MACS kit (130-092-657, Miltenyi Biotec, Germany). These NK cells were co-cultured with HepG2 or HepG2.2.15 at effector to target ratio of 1;1. After co-culturing for 4 hours, NK cells were stained with CD3 and CD56 monoclonal antibody. After staining, methanol (100μl/well, 15 minutes) and a fixation/permeabilization solution (554714, BD Bioscience, 100μl/well, 15 minutes) were added. After fixation, the samples were stained with anti-human pSTAT1 and pSTAT4 monoclonal antibody and analyzed using flow cytometry. *; P <0.05. (TIF) [file pone.0174103.s004.tif]

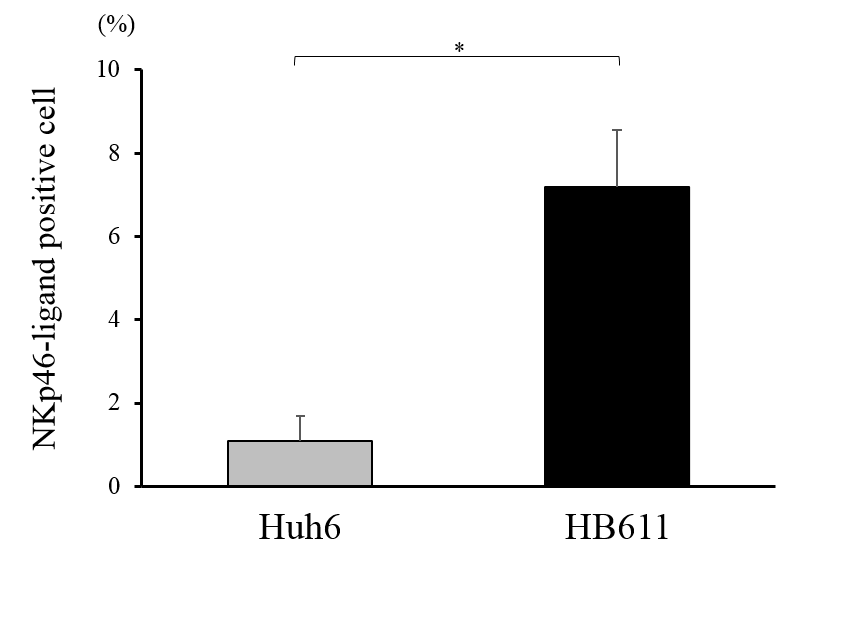

Supplement: S4 Fig — The expression of NKp46-ligand in Huh6 and HB611 were analyzed by flow cytometry. The method was mentioned in Patients and method. *; P <0.05. (TIF) [file pone.0174103.s005.tif]

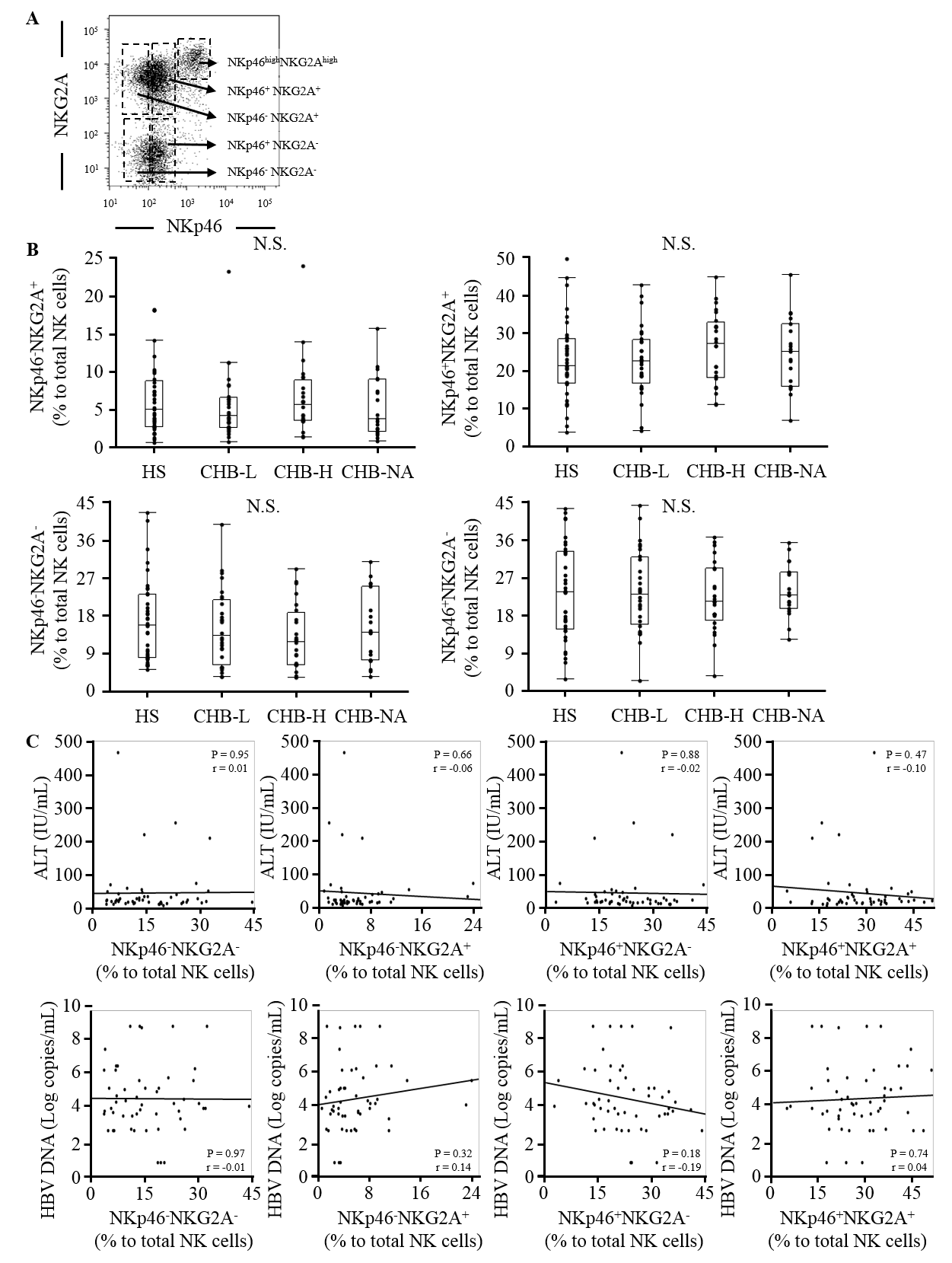

Supplement: S5 Fig — (A) CD56+CD3- NK cells were classified into NKp46highNKG2Ahigh, NKp46-NKG2A-, NKp46+NKG2A-, NKp46-NKG2A+ and NKp46+NKG2A+ subset. The borderline of NKp46 was determined by isotype control (as shown in S2A Fig.). (B) The frequencies of NKp46-NKG2A-, NKp46+NKG2A-, NKp46-NKG2A+ and NKp46+NKG2A+ subset were assessed among 108 patients consisted of 35 HS, 28 CHB-L, 24 CHB-H, 19 CHB-NA. (C) Linear regression analysis between the frequencies of these NK cell subsets and serum ALT or HBV DNA levels. The lines represent regression lines. (TIF) [file pone.0174103.s006.tif]
